# Physical activity and dose-characteristics in free-living conditions among patients awaiting coronary artery bypass graft surgery: a cross-sectional study

**DOI:** 10.1186/s12872-025-05136-2

**Published:** 2025-09-30

**Authors:** Sandra Dijkstra, Setayesh R. Tasbihgou, Iris Tigchelaar, Sawal D. Atmosoerodjo, Rolf Huet, Lucas H.V. van der Woude, Massimo A. Mariani, Anthony R. Absalom

**Affiliations:** 1 Department of Cardio-Thoracic Surgery, University of Groningen, University Medical Center Groningen, Hanzeplein 1, Groningen, Groningen, 9713 GZ the Netherlands; 2 Department of Anesthesiology, University of Groningen, University Medical Center Groningen, Groningen, the Netherlands; 3 Department of Ophthalmology, University of Groningen, University Medical Center Groningen, Groningen, the Netherlands; 4 Center for Human Movement Sciences, Department of Rehabilitation Medicine, University of Groningen, University Medical Center Groningen, Groningen, the Netherlands

**Keywords:** Exercise, Cardiac surgical procedures, Accelerometry, Prevention, Preoperative period, Self report, Fitness trackers, Adult, Activities of daily living, Healthy lifestyle

## Abstract

**Background:**

Physical activity prior to elective coronary artery bypass grafting (CABG) surgery can potentially improve postoperative outcomes. The aim of the study was to evaluate subjectively and objectively physical activity levels and dose-characteristics in free-living conditions in patients awaiting CABG surgery.

**Methods:**

A single-centered cross-sectional subanalysis of 32 participants awaiting elective CABG surgery. Physical activity during the preoperative period was assessed subjectively with the SQUASH questionnaire and objectively with the Sensewear activity monitor (accelerometer with physiological sensors). The Wilcoxon signed-rank test was used to assess differences and Bland-Altman plots were used to assess the agreement between the measurement methods. Descriptive statistics were used for the dose-characteristics and Cohen’s Kappa for the proportion of participants fulfilling the Dutch guideline for healthy physical activity (NNGB).

**Results:**

Duration of vigorous activity was significantly higher when measured subjectively (120 min/week) than objectively (7 min/week, *p* = 0.001). Bland-Altman plots showed that differences between the methods increased with longer durations of moderate and total activity. The dose-characteristics of physical activity measured with the SQUASH varied widely among the participants and it consisted mainly of leisure time activities and light household activities. The percentage of participants complying with the NNGB guideline was 74% when measured subjectively and 90% when measured objectively (κ = 0.259, *p* = 0.089).

**Conclusions:**

Surprisingly, both measurement methods suggest that the majority of patients awaiting CABG surgery met the recommendations of the NNGB guideline. The agreement between the methods however decreased with higher physical activity levels. Despite its limitations, the study suggests the complementary value of a subjective and objective measurement of physical activity in free-living conditions among CABG patients.

## Background

Patients presenting for coronary artery bypass grafting (CABG) surgery are commonly thought to have an unhealthy and inactive lifestyle [1–3]. In addition, and possibly because of lifestyle risks, these patients usually have a pre-existing low-level baseline inflammatory state [4]. This may predispose them to an exaggerated inflammatory response to surgical insults, thereby increasing the risk of postoperative cognitive, cardiovascular, and pulmonary dysfunction [4, 5]. Physical activity (PA) is defined as any bodily movement produced by skeletal muscles that results in energy expenditure [6]. PA before surgery, also as implemented in preoperative rehabilitation programmes (prehabilitation), can indeed help to attenuate the inflammatory state and to optimise the physiological reserves for surgery, and thereby improve postoperative outcomes and recovery [4, 5, 7–9]. Levels of PA and its dose-characteristics are largely unknown in this population [10], yet are deemed important from an exercise physiological and health perspective in the initiation and planning of preoperative rehabilitation or other preventive measures for patient groups as well as at the individual level [11].

Multiple types of assessments for measuring the level of PA are used depending on financial and research resources [12, 13]. Questionnaires are the preferred strategy to assess PA in larger populations and have also been used in studies of PA prior to cardiac surgery [13–18]. The Short QUestionnaire to ASsess Health-enhancing physical activity (SQUASH) is a reliable and validated tool that is widely used to assess PA in the Netherlands [19]. While self-report measures like the SQUASH are easy to implement, offer a very low burden to participants, and are relatively inexpensive, they are thought to be subject to recall bias [12, 13].

Highly accurate objective methods do exist, including the doubly labelled water (DLW) method which is considered the ‘gold standard’ for measuring levels of energy expenditure (EE) of PA during free-living conditions [12, 13]. These objective methods are, however, very expensive and impractical for clinical research.

Alternatively, portable activity monitors offer a more practical method of objective PA assessment [12, 13]. Their advantage is that they provide information about PA in free-living conditions, while remaining less expensive than the highly accurate objective methods and are less vulnerable to responder bias associated with questionnaires. Nonetheless, activity monitors can be more burdensome for subjects because they need to be worn for multiple days. The Sensewear (Bodymedia, Pittsburgh, PA, USA) is a portable activity monitor that has been shown to accurately estimate total energy expenditure for older adults in free-living conditions in comparison to the DLW method [20–22]. Accelerometers like the Sensewear have been used for PA monitoring in a variety of cardiac patient groups, especially for assessing the effect of postoperative cardiac rehabilitation, but little is known about PA levels during regular daily life in patients awaiting cardiac surgery [21, 23–28]. To our knowledge, no study has yet evaluated PA levels in free-living conditions in patients awaiting CABG surgery measured both subjectively via a questionnaire and objectively with an accelerometer.

People with cardiovascular problems, especially those awaiting CABG, are expected to be less physically active than the overall population. The Dutch guideline for healthy PA (‘Nederlandse Norm Gezond Bewegen’ NNGB) is similar to the recommendations from the World Health Organization and other national recommendations, and recommended that adults should be physically active with moderate intensity for at least half an hour on at least five days per week, but preferably every day [29].

Questionnaires and monitors for assessing PA, like the SQUASH and Sensewear, have been designed and validated in the healthy population, but not yet in cardiac surgery patients who are likely to respond to PA differently due to their limited cardiac reserve. The first aim of this cross-sectional study was to evaluate the levels of PA in free-living conditions subjectively by using the SQUASH questionnaire and objectively by using the portable activity monitor Sensewear among patients awaiting CABG surgery. Secondary objectives of the study were to evaluate the dose-characteristics of PA in this patient group and compare the results of both methods to the Dutch guideline for healthy physical activity (NNGB). The results of this study could offer insight into the expectations of preoperative rehabilitation (prehabilitation) programmes for this patient group [30].

## Methods

### Design overview

This study is a cross-sectional subanalysis of data nested within the ‘Sarcopenia study’. The Sarcopenia study was a single-centered longitudinal observational study analysing levels of perioperative PA and postoperative outcomes in CABG patients. Details of the study protocol, including the sample size calculation, are described elsewhere [31]. The study was approved by the medical ethics committee (METC: reference number 2014/219) of the University Medical Centre Groningen (UMCG), the Netherlands, and all included participants gave written informed consent. All participants in the Sarcopenia study were asked to complete the SQUASH questionnaire and to undergo assessments for mental, cognitive, and physical health. There are no guidelines for physical activity in patient awaiting elective CABG surgery in the Dutch hospital environment. Patients awaiting CABG surgery were generally advised to continue their usual activities as much as possible without triggering symptoms. Patient and surgical characteristics, including blood samples, were routinely collected in surgical databases as part of usual care procedure.

After the start of the study, the ethics committee approved an application to amend the protocol to allow use of the Sensewear monitor as well. The current study only involved the data of the participants who used the Sensewear monitor and received their operations between December 2015 and March 2017. The investigation conforms with the principles outlined in the Declaration of Helsinki.

### Participants

Patients scheduled for elective coronary artery bypass grafting surgery at the UMCG were included in this study. Recruitment took place during the preoperative screening after the consultation with the anaesthesiologist. Further inclusion criteria of the Sarcopenia study were that participants must have been above 18 years old, had to be able to stand and walk independently and perform the tests of physical fitness and cognitive functioning.

Exclusion criteria as stated by the Sarcopenia study protocol were a combination of CABG with a valve procedure or reoperations, if postoperative intensive care stay was expected to be extended, or a recent history of depression or severe anxiety, or a history of cognitive impairment (i.e., dementia, other neurological disorders, stroke, or other severe cerebrovascular insults). Patients were excluded if they were unable to understand or read Dutch instructions.

### Self-reported physical activity

Participants were asked to fill in the Short QUestionnaire to ASsess Health-enhancing physical activity (SQUASH) after their preoperative anaesthetic screening visit. It is a standardised self-reported recall questionnaire which consists of both open and closed questions to assess the dose-characteristics (i.e., frequency, the duration, and the perceived intensity) of several activities of an average week in recent months [19]. There are four recordable domains of activity in the SQUASH: commuting, activities at work, household activities, and leisure time. Outcome measures of the SQUASH questionnaire were the durations of PA in the intensity categories (min/week, light, moderate, and vigorous), dose-characteristics of PA, and the proportion of participants fulfilling the NNGB guideline. Dose-characteristics for each activity domain were evaluated in terms of frequency (times/week), duration (min/week), intensity score, and activity score.

#### Data analysis self-reported physical activity

A syntax file for IBM SPSS statistical package (version 22, SPSS, Inc., Chicago, Illinois, U.S.A.) was used to evaluate the outcome measures of the SQUASH questionnaire, as per Wendel-Vos et al., 2003 [19]. We applied the classification used in Ainsworth’s compendium of physical activities to assign each activity reported in the SQUASH questionnaire a metabolic equivalent of task (MET) score [32]. With the MET cut-off scores the activity was assigned to an intensity category (Table 1). The time spent on activities in the specified intensity categories and in the specific activity domains were summed. Time spent in total activity was the summation of moderate and vigorous activity.

**Table 1:** Validated cut-off scores for determining the levels of physical activity using the SQUASH questionnaire and the Sensewear monitor

Age	Light/Sedentary^a^		Moderate		Vigorous	
	SQUASH	Sensewear	SQUASH	Sensewear	SQUASH	Sensewear
18–55 years	< 4 MET	< 4 MET	4–6.5 MET	4–6 MET	≥ 6.5 MET	≥ 6.0 MET
> 55 years	< 3 MET	< 3 MET	3–5 MET	3–6 MET	≥ 5 MET	≥ 6.0 MET

*Abbreviations* *MET* metabolic equivalent of task

^a^SQUASH only light activities, Sensewear combines light activities and sedentary time

Furthermore, an intensity score was given to the activity in each domain by a combination of the perceived intensity reported by the participants in the questionnaire and the intensity category based on Ainsworth’s compendium [32]. The intensity score was 1–3 MET for light, 4–6 MET for moderate, and 7–9 MET for vigorous level according to the compendium, sequentially from light, moderate to vigorous according to the perceived intensity. In addition, an activity score was computed by multiplying the total minutes of the activity with its intensity score. Finally, we determined whether the PA of individual participants met the NNGB guideline in existence at the time. The NNGB recommended that adults should be physically active with moderate intensity for at least half an hour on at least five days per week, but preferably every day [29]. This may be considered as 150 min/week, which is the duration recommended by the current World Health Organization (WHO) guideline [10]. Thus, we considered participants to have met the NNGB guideline if they reported that they had been moderately or vigorously active for at least 150 min/week.

### Portable physical activity monitor

After the preoperative anaesthetic screening visit, participants were given the Sensewear Mini Armband Model MF-SW (Bodymedia, Pittsburgh, PA, USA). They were given verbal and written instructions about the use of this device. The device contains multiple sensors of acceleration in three axes, heat flux, skin temperature and galvanic skin response. The Sensewear has been validated for estimating daily energy expenditure compared with indirect calorimetry and DLW method [20–22]. Data was recorded at a standard epoch length of 60s. Participants were instructed to wear the monitor on the back of the left upper arm in free-living conditions for three consecutive days, also during the night, leaving it off one hour per day and removing it to perform water-based activities. Participants received no instructions on which specific days the device should have been worn. The device had no on/off button and turned on and began collecting data within ten minutes of being worn. Participants were asked to fill in an additional daily logbook about their physical activities during the days that the device was worn. The Sensewear was used to determine which participants fulfilled the NNGB guideline, the durations of PA in the intensity categories (min/week, sedentary, moderate, and vigorous) and total energy expenditure (kcal).

#### Data analysis portable physical activity monitor

The Sensewear software (SenseWear 8.1, Bodymedia, Pittsburgh, PA, USA), which takes account of patient characteristics (i.e., age, gender, weight, height, handedness, and smoker/non-smoker), determined the amount and intensity of PA during each day (Table 1). Only valid days, defined as days when the Sensewear was worn for more than ten waking hours, were included in the analysis. The durations of PA in the intensity categories during waking hours and total energy expenditure of the entire day were calculated, averaged over the valid days, and extrapolated to a whole week (multiplied by seven) in MATLAB (version R2014a, Mathworks, Natick, Massachusetts, U.S.A.). Subsequently, the data in MATLAB was exported to a Microsoft excel file (version 2010, Redmond, WA, U.S.A) which was imported into the SPSS database. The duration of total activity was the summation of moderate and vigorous activity. The total duration of moderate and vigorous activity per participant was used to determine whether the Sensewear data indicated that the PA levels of the participant met the NNGB recommendations.

### Statistical analysis

Descriptive statistics of all relevant variables were calculated and analysed with SPSS software (version 22, SPSS, Inc., Chicago, Illinois, U.S.A.). Paired samples t-tests were carried out to assess differences between the two methods measuring moderate, vigorous, and total physical activity. When the data were not normally distributed, the non-parametric equivalent, the Wilcoxon signed-rank test, was used. Bland-Altman plots were prepared with 95% limits of agreement to illustrate the agreement between the two methods for the PA levels [33]. Cohen’s Kappa (κ) was used to assess the agreement between the two methods in determining compliance with the NNGB guideline. Two-sided statistical tests were performed and the results considered significant at an alpha of 5%.

## Results

### Participants

**Fig. 1:**
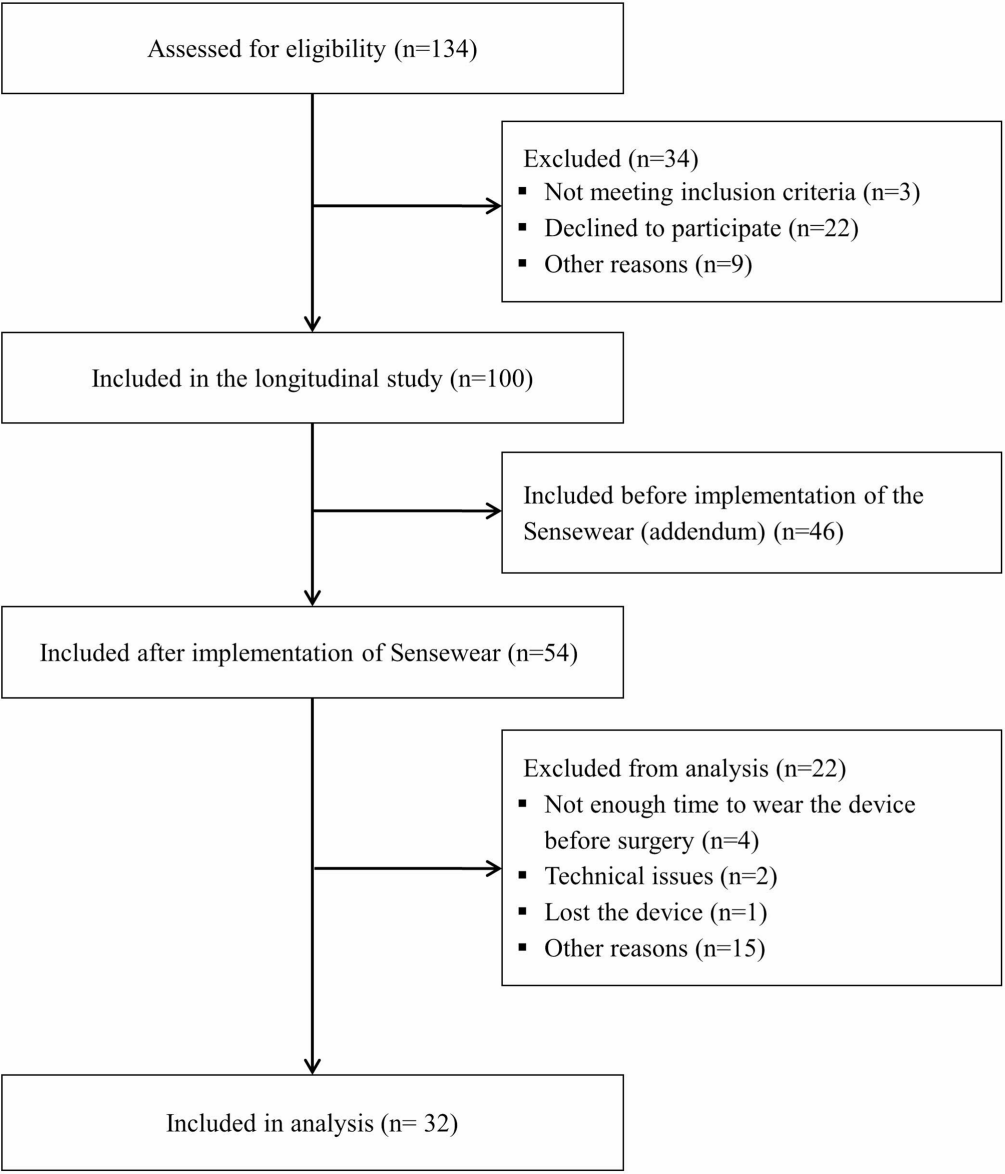
*Flow diagram of participants included in the longitudinal Sarcopenia study* [31] *and included in the study*

Characteristics of the participants included in this cross-sectional subanalysis are summarized in Table 2 and the flowchart of the included participants and reasons for exclusion are shown in Fig. 1. In one participant the planned CABG procedure was cancelled. The SQUASH questionnaires were completed 28 ± 13 days before surgery. The Sensewear was worn starting 25 ± 13 days before surgery. Sensewear data were available for 59% of the 32 participants included in this subanalysis. Participants wore the Sensewear for an average of 3.8 ± 0.7 days, 1052 ± 150 min a day, 752 ± 159 min when being awake. The mean number of valid days (> 10 h during waking hours) was 2.8 ± 0.6 days. 55% of the participants wore the device during the weekend, and of these participants, 70% wore the device on both weekend days. Of all days we considered valid days of Sensewear use, 32% were weekend days. As a weekend represents 2/7 (29%) of a week, we consider that weekend and week days are proportionally well represented. One participant was excluded from the analysis because the measurement did not contain any valid days. One participant observed skin irritation after wearing the Sensewear but was able to complete the assessment.

**Table 2:** Patient characteristics (*n* = 32)

	CABG patients
Age (years)	65 ± 9
Gender (male)	29 (91)
Height (m)	1.77 ± 0.07
Weight (kg)	84 ± 14
BMI (kg/m^2^)	27 ± 3
Handedness (Right-handed)	31 (97)
Hypertension	20 (63)
Diabetes Mellitus	7 (22)
COPD	4 (13)
Smoking	5 (16)
Logistic EuroSCORE (*n* = 31)^a^	4.86 ± 5.33
Preoperative inflammatory markers	
CRP (mg/l)	4 ± 6
Leukocytes (×10^9^/l)	8 ± 2
Thrombocytes (×10^9^/l)	246 ± 56

Data are presented as mean ± SD or as the absolute *n* (%)

*Abbreviations* *BMI* Body Mass Index, *CABG* coronary artery bypass grafting, *COPD* chronic obstructive pulmonary disease, *CRP* C-reactive protein

^a^one participant eventually did not undergo surgery

### Physical activity levels

**Fig. 2:**
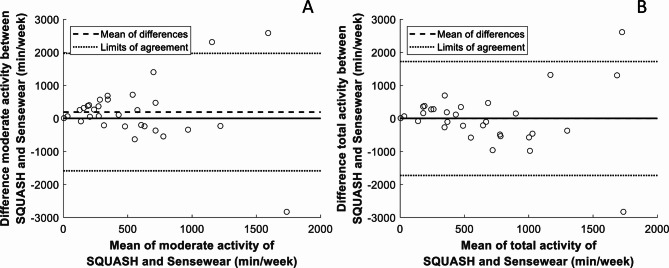
Bland-Altman plots for moderate (**A**) and total (**B**) physical activity (min/week) measured by the self-reported measure, the SQUASH questionnaire, and the arm worn physical activity monitor, the Sensewear, of the patients awaiting coronary artery bypass grafting (*n* = 31)

The durations of PA in the intensity categories as assessed by the two methods are shown in Table 3. The durations of vigorous activities registered by the methods were significantly different. Figure 2 shows that most data points lay between the limits of agreement in the Bland Altman plots, but that differences between the assessments increased with longer durations of activity. Light activities reported via the SQUASH questionnaire had a median [range] duration of 828 [10-3360] min/week. Participants were sedentary according to the Sensewear for a median [range] of 6090 [3885–7203] min/week. The mean ± SD total daily energy expenditure according to the Sensewear was 2350 ± 532 kcal.

**Table 3:** Physical activity prior to CABG surgery measured by the SQUASH questionnaire and the Sensewear monitor. (*n* = 31)

	SQUASH	Sensewear	*P* value^a^
Moderate (min/week)	180 [0-3150]	483 [7-2884]	0.092
Vigorous (min/week)	120 [0-1035]	7 [0-427]	0.001*
Total (min/week)	480 [0-3150]	490 [7-3031]	0.891

Data are presented as the median [range]

*Abbreviations* *CABG* coronary artery bypass grafting

^a^non-parametric Wilcoxon signed-rank test

^*^significant result *p*<0.05

### Dose-characteristics of physical activity

The evaluation of dose-characteristics determined from the results of the SQUASH questionnaire (Table 4) shows considerable diversity in context and forms of PA as well as in intensity, frequency, and duration among the participants. Moreover, it shows that 10% of participants had walked or cycled to work or school, and mostly only light activities were performed during work (26% of participants). The majority of the participants (84%) reported performing light intensity household activities (median [range] 210 [0-1680] min/week) and 26% of participants performed vigorous household activities (median [range] 0 [0-750] min/week). Leisure time activities mainly consisted of walking (71% of participants, median [range] 105 [0-1050] min/week) and playing sports (65% of participants, median [range] 170 [0-1260] min/week). Among those who engaged in sports, the median [range] estimated MET-value across all reported sports activities was 2.3 [1.3–6.0].

**Table 4:** Physical activity prior to CABG surgery in the four domains of the SQUASH questionnaire (*n* = 31)

Domain	Number of participants (*n*)	Frequency (days/week)	Duration per week (min/week)	Intensity score^a^	Activity score^b^
Commuting	3		0 [0-360]		0 [0-3240]
Walking	0	0	0 [0–0]	0 [0–0]	0 [0–0]
Bicycling	3	0 [0–4]	0 [0-360]	0 [0–9]	0 [0-3240]
Activities at work	9		0 [0-360]		0[0-3240]
Light	8		0 [0-2400]	0 [0–0]	
Intense	1		0 [0-3000]	5 [5–5]	
Household activities	26		240 [0-1800]		0 [0-3750]
Light	26	5 [0–7]	210 [0-1680]	0 [0–0]	0 [0–0]
Intense	8	0 [0–5]	0 [0-750]	5 [5–5]	0 [0-3750]
Leisure time	26		870 [0-1720]		[525–9310]
Walking	22	3 [0–7]	105 [0-1050]	4 [0–6]	600 [0-5250]
Bicycling	11	0 [0–7]	0 [0-630]	0 [0–8]	0 [0-4410]
Gardening	13	0 [0–6]	0 [0-780]	0 [0–8]	0 [0-6240]
Odd jobs	15	0 [0–7]	0 [0-720]	0 [0–6]	0 [0-2880]
Sports	20		170 [0-1260]		0 [0-2400]

Data are presented as the median [range] or *n*

*Abbreviations* *CABG* coronary artery bypass grafting

^a^Intensity based on a combination of perceived intensity and the Ainsworth compendium following Wendel-Vos et al., 2003

^b^Activity score = minutes/week x intensity score

### Dutch guideline for healthy physical activity (NNGB)

Analysis of the SQUASH questionnaire results revealed that 74% of the participants met the NNGB guideline for healthy physical activity, whereas the results from the Sensewear monitor suggested that 90% of the participants fulfilled the NNGB guideline. The agreement between the measurements had a Cohen’s Kappa value of 0.259 (*p* = 0.089).

## Discussion

The aim of this cross-sectional study was to evaluate the levels of PA and dose-characteristics in free-living conditions by using a subjective and objective method in patients awaiting CABG surgery. For vigorous levels of PA, the self-reported duration was much higher than the objectively measured duration. In general, the objectively measured duration of vigorous activity was minimal. Differences between the assessments increased with longer durations of moderate and total activity. The dose-characteristics of PA varied widely among the participants and it consisted mainly of leisure time activities and light household activities, and almost no activities during commuting and work. A non-significant, fair agreement was observed between the two methods for determining whether participants met the NNGB guideline.

Little is known about PA levels prior to CABG surgery, especially when assessed with activity monitors in free-living conditions [14, 15]. In this study, the majority of participants met the criteria of the NNGB guideline before surgery. Interestingly, the proportion of the general Dutch population who complied with the NNGB guideline was lower than the percentage than we found among our patients, regardless of whether it was assessed with the SQUASH questionnaire or the Sensewear [34]. This was contrary to our expectation that CABG patients would have lower levels of PA, since inactivity, among others, is a risk factor for arteriosclerosis.

This finding may be associated with the appreciation and perceptions of patients of their PA in the context of a potentially reduced physical work capacity, fear of exercise-induced chest pain, or breathlessness, and their tendency to higher levels of perceived fatigue, which will be different from those in the healthy population [35]. It is notable that the Sensewear data, which was an objective assessment, also showed high levels of total PA. This might be because the Sensewear also measures heat flux, skin temperature and the galvanic skin response in addition to monitoring movement, and these measurements provide an objective assessment of the influence of the activity on the cardiovascular system, showing higher activity intensities for activities that are less demanding on healthier patients. Thus, cardiac patients could have experienced their exercise as more intense, they actually performed more PA compared to the general Dutch population, or both.

Although total physical activity appears high for this patient group, total energy expenditure was lower than that reported in healthy adults using the DLW method [36]. It is possible that after bouts of moderate or vigorous exercise, participants were more sedentary and/or resting causing a reduction in their total daily energy expenditure. Total energy expenditure might be a more appropriate measure for the level of PA throughout the day, consistent with the updated WHO guidelines that stated that every bout of PA is relevant, including those lasting less than 10 min [10]. However, it is not possible to capture total energy expenditure using questionnaires, the method preferred for evaluation of the behaviour of larger populations. Participants in our study were sedentary for the majority of the day, which is similar to the findings of other studies of patients undergoing cardiac rehabilitation [24, 25]. As a sedentary lifestyle increases the risk of postoperative complications and mortality, to limit the amount of time that patients are sedentary might be worthwhile in this patient group [37]. The updated WHO guidelines also shows the importance of the whole spectrum of PA, implying that a decreasing time spent sedentary by spending a longer duration on light activities is also beneficial [38]. Nonetheless, more research on PA among specific patient populations, such patients requiring cardiac surgery, is needed [38].

Although subjective and objective methods showed high levels of PA in our study, the agreement between these two methods was rather low. The results of the Bland-Altman plots show that when PA increases, for both moderate and total physical activity, the agreement between the two methods decreases. These results seem to be consistent with other research which found that self-reported measures tend to overestimate PA levels compared to objective measures [14, 21]. An explanation for the lower levels of agreement may be that the validated methods use different data processing techniques and MET cut-off scores. Furthermore, these underlying algorithms are largely based on studies in healthy individuals [15, 21, 23]. A trend can be observed where the technology of PA devices is improving, but validation studies remain scarce, particularly studies involving patient specific algorithms that could potentially have higher validity than algorithms designed for healthy populations [39]. A study using the Sensewear in healthy adults found that it underestimated higher intensities of PA, but it is unknown if this also applies to patients with cardiovascular problems [22]. Both the perceived and the objective PA construct require validation among specific patient groups, including ours, and should be updated with the most recent available data [39].

Little is known about PA behaviour in patient populations in terms of dose-characteristics (i.e., context, form, duration, intensity, frequency). It is assumed that both the subjective SQUASH and the objective Sensewear methods have their value, largely complementary to one another. This complementary value has been emphasized in a recent review, which notes that none of the device-based PA tools can fully capture the complete construct of PA, including its dose-characteristics [39]. Indeed, the Sensewear provides an objective intensity and duration indicator of accelerometry-based PA and energy expenditure throughout the day in free-living conditions, while the SQUASH provides us estimates for similar gross measures, but additionally provides information on context and form-dependent indicators of PA in intensity, frequency, and duration.

In addition to the aforementioned challenges in assessing PA in free-living conditions in patients awaiting elective CABG surgery, these patients may be at risk during exercise and seem to experience exercise as more intense than the healthy population. Therefore, it is important that these patients can exercise in a safe environment, for example in a structured exercise programme in a rehabilitation centre. Furthermore, such a prehabilitation programme may improve their physical functioning and recovery from surgery [8, 9, 40].

In the time since our study data were collected, research on preventive measures and prehabilitation has gained increasing attention [8], which may have also shifted patients’ perspectives on PA before surgery over recent years. It is possible that patients are currently more physically active than in 2015–2017. Indeed, the percentage of individuals adhering to physical activity guidelines has increased over the last decades, both in the general population in the Netherlands and among patients with cardiovascular disease in the United States, although this increase has been minimal [34, 41]. Given those trends, the impact of attempts to implement prehabilitation programmes (which is still not common) probably did not significantly affect current PA behaviour in daily life. Our results can be interpreted as a baseline understanding of PA behaviour during regular daily life in patients awaiting cardiac surgery.

Baseline dose-characteristics of PA in daily life at the group level help understand the construct of PA in patients, are useful for evaluating individual scores and to determine the dose-characteristics needed to tailor structured PA in prehabilitation [11]. Currently there are no guidelines for preoperative exercise for patients awaiting cardiac surgery, so it is important to evaluate their exercise performance during the programme both subjectively and objectively in order to evaluate the dose-response characteristics in patients prior to CABG [30]. Activity related monitoring and exercise technology may help the development of home-based rehabilitation care, both pre- and postoperatively [8]. In the context of growing healthcare demand in the fact of limited and finite personnel and financial resources, such home-based rehabilitation may be cost-effective, particularly if combined with some form of remote supervision or monitoring. Patient safety and supervision are critical here.

Some additional limitations of the study mentioned. Among these limitations is the small sample size combined with the single-centre design, which limits both statistical power and generalizability. Another is selection bias, as it is possible that the more physically active patients agreed to be included in the study. A form of observation bias is also possible, as wearing of the monitor may have reminded participants of the need for physically activity. In addition, the SQUASH questionnaire was completed prior to the period when the Sensewear was worn, and therefore refers to a different period of time. As is usual practise in our institution, patients only have one preoperative anaesthetic screening visit, and it was therefore during this visit that participants were given instructions on how to fill in the questionnaires and use the Sensewear. This probably led to a limited overlap of the two time windows of measurement in each of the two instruments, which is not uncommon in similar studies [21]. Nonetheless, it would have provided more robust results if the two instruments had been used over exactly the same time period. Also, a longer duration of data collection with the Sensewear monitor (at least 7 days) combined with a logbook documenting specific activities and timestamps, would have provided a better time sample of PA. This approach could have improved our understanding of the average PA for a whole week including both week and weekend days. Despite these (common) limitations in the measurement time frames of both techniques, comparing the two from different perspectives, under the assumption that they both measure complementary aspects of PA, is still valid and helps bridge our lack of understanding of PA (and their dose-characteristics) in daily life among patients awaiting cardiac surgery.

## Conclusions

In a cohort of patients awaiting CABG surgery, both subjective and objective measures of PA suggested that the majority of CABG patients fulfilled the NNGB guideline. However, the agreement between the subjective and objective methods decreased with higher levels of PA. This study suggests that the two methods used in this patient group complement each other, since the SQUASH provides information on context and form-dependent indicators of PA and the Sensewear provides objective intensity and durations of PA and its energy expenditure. Future research should involve a larger group of cardiac surgery patients to determine preoperative levels and the dose-response relationship of PA in order to further help specify physical activity guidelines in this patient group.

## Data Availability

All data generated or analysed during this study are included in this published article [and its supplementary information files].

## Abbreviations

CABG: Coronary artery bypass grafting
DLW: Doubly labelled water
EE: Energy expenditure
MET: Metabolic equivalent of task
NNGB: The Dutch guideline for healthy physical activity
PA: Physical activity
SQUASH: Short QUestionnaire to ASsess Health-enhancing physical activity
UMCG: University Medical Centre Groningen
WHO: World Health Organization
